# Differences in experiences of patients with advanced cancer in Japan from 3 to 6 years after diagnosis

**DOI:** 10.1007/s11764-025-01761-0

**Published:** 2025-02-13

**Authors:** Yuichi Ichinose, Tsutomu Toida, Tomone Watanabe, Takafumi Wakita, Takahiro Higashi

**Affiliations:** 1 Department of Public Health and Health Policy, Graduate School of Medicine, The University of Tokyo, Hongo 7-3-1 Bunkyo-Ku, Tokyo, 113-0033 Japan; 2 Division of Health Services Research, Institute for Cancer Control, National Cancer Center, Tsukiji 5-1-1 Chuo-Ku, Tokyo, 104-0045 Japan; 3 Faculty of Economics, Dokkyo University, Gakuen-Cho 1-1 Soka-Shi, Saitama, 340-0042 Japan; 4 Faculty of Sociology, Kansai University, Yamate-Cho 3-3-35 Suita-Shi, Osaka, 564-8680 Japan

**Keywords:** Neoplasm, Survey, Quality of life, Patients, Family

## Abstract

**Purpose:**

Coping with cancer presents significant challenges, especially for those with advanced-stage and long-term survival. However, research on advanced-stage cancer experiences in Japan remains limited. This study analyzed how patient experiences with advanced-stage cancer/long-term survival varied across different diagnosis periods.

**Methods:**

We examined two groups of patients with advanced cancer diagnosed in 2013 and 2016 using data from the Patient Experience Survey, a nationwide survey of cancer patients in Japan in 2019. Weighted analysis was used to estimate the distribution of patient experiences in the representative population. We compared the experiences of patients diagnosed with advanced-stage disease in 2016 (newer diagnosis group) and 2013 (earlier diagnosis group).

**Results:**

We analyzed 1584 participants in the newer diagnosis group and 412 in the earlier diagnosis group, with response rates of 30.8% and 43.0% respectively (*P* < 0.01). The earlier group had more proxy responses (38.0% vs. 43.2%). Survey response distribution was similar across groups; however, earlier diagnosis patients reported worse access to treatment information, lower satisfaction, and less positive post-treatment experiences than did newer patients. However, when considering respondent type, patient responses were consistent across years, whereas proxy responses were more negative.

**Conclusions:**

Differences in survey timing and response types significantly impact the reported patient experiences. Policymakers should consider these factors when designing cancer control strategies to support patients and families.

**Implications for Cancer Survivors:**

Policymakers should use these findings to enhance cancer control strategies, addressing the distress of patients and families affected by advanced and long-term cancer.

**Supplementary Information:**

The online version contains supplementary material available at 10.1007/s11764-025-01761-0.

## Introduction

Cancer continues to be a burden for individuals even after their treatment concludes. The overall incidence of cancer is approximately 19.3 million globally, with 1 million cases reported in Japan [1, 2]. The combination of a rapidly super-aging society and the increasing relative survival rate of cancer survivors owing to early diagnosis and improved treatment have resulted in an increase in the number of patients with cancer [3]. Moreover, the need for cancer care among patients with cancer varies depending on the individual’s treatment process or social situation. Studies suggested that patients who achieved long-term survival underwent adjustments in physical, psychological, and social dimensions [4–7]. However, other studies reported that patients with advanced cancer have a lower quality of life (QoL) and experience more symptoms [8–10]. Moreover, these long-term survivors continued to contend with cancer and cancer-related issues, such as anxiety about recurrence or financial difficulties [11]. Although ongoing care for cancer patients is necessary, studies on the experience of patients with long-term and/or advanced cancer are scarce in Japan. Some studies followed up with patients with long-term and advanced cancer; however, their sample size is limited because they used cohorts recruited from some municipalities or hospitals in Japan [12, 13].

To monitor the progress of the Cancer Control Plan from the patients’ perspectives in Japan, the National Cancer Center conducted a Patient Experience Survey (PES). The PES recruited patients with cancer from all the prefectures in Japan, and the results of the PES can project the representation of patients with cancer in Japan using a probability sampling design. We originally recruited patients diagnosed with cancer 3 years prior in the first PES. Because patients with long-term/advanced cancer might report different experiences, we included patients diagnosed 3 and 6 years prior in the second PES.

Using the samples from the PES, we selected two groups of patients with advanced-stage cancer diagnosed at different times (2013 or 2016). We aimed to compare the demographics of patients who were diagnosed earlier or later and to assess any differences in cancer patients’ experiences of patients with different diagnosis years.

## Patients and methods

### Study design

The PES was a nationwide cross-sectional survey in Japan, using a self-administered questionnaire administered to cancer patients in 2019. For the main survey, patients diagnosed in 2016 were recruited; detailed methods and results have been reported elsewhere [14]. In addition, for comparison purposes, patients diagnosed with stage III-IV diseases in 2013 were surveyed from the same participating hospitals and thus analyzed in this study.

This study was approved by the Institutional Review Board of the National Cancer Center (2018–218). Informed consent was obtained from all the participants at the beginning of the survey.

### Source population

We obtained samples from the Hospital-Based Cancer Registry (HBCR) as the source population. The HBCR is a mandatory cancer case-reporting system for all designated cancer care hospitals and many non-designated cancer care hospitals in Japan. The designated cancer care hospitals are selected by each prefecture and designated by the Ministry of Health, Labour, and Welfare. The HBCR aims to collect detailed cancer information, such as basic demographic information, tumor location, histological information, cancer treatment, and other information based on standardized rules. Compared to the national incidence statistics, the HBCR accounts for approximately 70% of all patients with cancer in Japan [15]. The HBCR is leveraged by hospitals and government authorities to improve the quality of cancer care and inform cancer control policies through data analysis. We used the HBCR as the source population because it can be de-identified at each facility, and more detailed cancer information was collected, such as identifying rare cancers based on the ICD-O-3.1 code.

### Sampling

Participants in the PES were randomly selected from patients registered in the HBCR. The PES utilized stratified two-stage random sampling among all the cancer patients registered in the HBCR. First, hospitals from each prefecture were selected. The number of selected hospitals varied depending on the type of accreditation status of the designated cancer hospitals. Then, we randomly selected cancer patients who were 19 years old or older from the selected hospitals at the time of diagnosis. Participants in the PES were stratified into four groups before random selection, focusing on age, rare cancer types, and cancer stage [16]. This study specifically analyzed patients with advanced-stage cancer (stage III-IV) diagnosed in 2013 and 2016. While other stratified groups were included for broader survey purposes, only patients with advanced cancer were randomly selected for the current analysis. Questionnaires were sent to the selected patients via mail. Participants who consented to participate in the study returned their anonymized answers (Supplementary Fig. 1).

### Study participants

We selected all patients diagnosed with stage III-IV cancer in 2013 and 2016 from the PES. Although the main body of this survey targeted patients diagnosed 3 years prior to the survey, it also sampled patients diagnosed 6 years prior to elucidate the views of patients with longer-term experiences post-diagnosis. Subsequently, we divided the patients into two groups: those diagnosed with stage III-IV cancer in 2016 (referred to as the “newer diagnosis group,” diagnosed with advanced cancer 3 years prior) and those diagnosed with stage III-IV cancer in 2013 (referred to as the “earlier diagnosis group,” diagnosed with advanced cancer 6 years prior). In this study, we focused on patients with stage III-IV cancer because patients with advanced-stage cancer tend to continue treatment for longer periods.

### Questionnaire and analysis variables

The PES questionnaire has been illustrated in previous literature [14]. We defined outcome variables for each question based on the following rule. As for questions with the five Likert-scale choices (Q13, Q18, Q24, Q28, Q33, and Q34), we calculated the percentage of positive responses combining “Agree strongly” and “Agree.” To avoid the ceiling effect, we treated the “Agree somewhat” response as nonpositive. For Q8 and Q9, we calculated the percentage of less than 1 month by diagnosis or starting treatment by combining the response “Less than 2 weeks” and “More than 2 weeks and less than 1 month.” For Q31 and Q32, the responses “very familiar” and “somewhat familiar” were treated as outcome responses. For questions allowing multiple responses, we calculated the percentage excluding those who chose the specific response (“None of the above happened” for Q17, “None of above” for Q25, and “I did not experience any of the above” for Q27). For other questions permitting a single response, we determined the proportion of specific replies as outcome responses (“I talked to someone” for Q10; “Yes” for Q11, Q12, Q16, Q19, Q22, Q23, Q26, Q29, and Q30; “Yes, I was able to” for Q20). Finally, we calculated the mean and standard deviation for these questions using the global scale, Q21.

### Statistical analysis

We developed a set of sample weights based on the sampling design and used them to estimate the distributions of patient experiences in the representative population. Operationally, the weights for each patient were calculated as the inverse of the probability of being sampled, and non-response was incorporated by assuming that all non-respondents occurred randomly.

First, we compared the experiences of patients diagnosed in 2013 and 2016, including patient and proxy responses. We then performed a detailed analysis comparing patient and proxy responses, focusing on the responses of the patients themselves. The questionnaire items covered aspects before, during, and after treatment. We analyzed participants’ responses to each question (Q8–Q13, Q16–Q34). We excluded the results of Q14 and Q15 because of their small sample sizes.

We calculated the frequency (proportion) for categorical variables and the mean (standard deviation) or median (interquartile range) for continuous variables. To compare the characteristics of the two groups, we conducted Rao and Scott corrections for the chi-square test [17]. Moreover, Welch’s *t*-test was conducted to compare the means of the two groups [18]. Although the two groups had different source populations (patients with cancer registered in 2016 and 2013), we assumed that they had similar patient backgrounds. In addition, the same survey method was used to obtain samples for each year of diagnosis. All statistical analyses were performed using R version 3.6.1 (R Core Team, Vienna, Austria), RStudio 2023.12.1 (RStudio Team, Boston, MA, USA), and Stata 17.1 (Stata Corporation, College Station, TX, USA) [19–21]. Statistical significance was set at *P* < 0.05.

## Results

### Overall characteristics

**Fig. 1:**
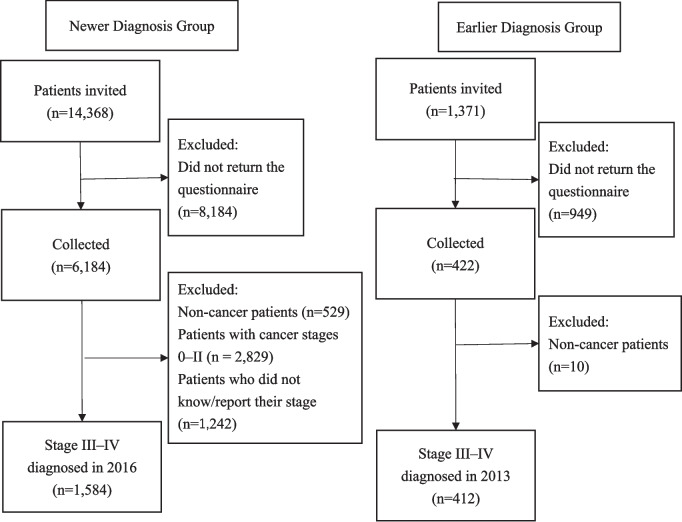
Flowchart of patient selection. The figure illustrates the flow of patient selection for the study

A total of 1996 patients were included in this study. The newer and earlier diagnosis groups comprised 1584 and 412 patients, respectively. The response rate in the earlier diagnosis group was lower than that in the newer diagnosis group (30.8% vs. 43.0%, *P* < 0.001). The details of patient selection are described in Fig. 1.

The baseline characteristics of the two groups are shown in Table 1. The most common cancer sites were the colon and lungs in patients with advanced cancer. Specifically, 21.4% of patients in the newer diagnosis group had colon cancer, and 17.9% had lung cancer, whereas in the earlier diagnosis group, 21.7% had colon cancer and 22.2% had lung cancer. The earlier diagnosis group had a higher percentage of proxy responses than did the newer diagnosis group (43.2% vs. 38.0%). The main reason for the proxy responses was that the patients had died at the time of the survey (83.7% in the newer diagnosis group and 91.7% in the earlier diagnosis group).

**Table 1:** Demographic characteristics of patients in the newer and earlier diagnosis groups

	Newer diagnosis group				Earlier diagnosis group			
	*N*	Percentage	§Corrected *N*	§Corrected %	*N*	Percentage	§Corrected *N*	§Corrected %
Sex								
Male	943	59.5%	59,434	60.7%	242	58.7%	78,086	53.5%
Female	634	40.0%	38,063	38.9%	167	40.5%	67,029	45.9%
No response	7	0.4%	394	0.4%	3	0.7%	793	0.5%
Age at survey, years								
Average (SD)	67.2	13.9	68.9	11.4	71.7	11.7	70.9	10.7
Median (IQR)	69	61–77	70	63–77	71	65–80	70	65–78
Respondents								
Patient	987	62.3%	60,238	61.5%	223	54.1%	82,726	56.7%
Family/other	590	37.2%	37,211	38.0%	188	45.6%	63,070	43.2%
No response	7	0.4%	441	0.5%	1	0.2%	113	0.1%
Self-reported cancer type								
Breast	119	7.5%	6029	6.2%	30	7.3%	13,448	9.2%
Colon	309	19.5%	20,940	21.4%	68	16.5%	31,667	21.7%
Stomach	171	10.8%	12,418	12.7%	50	12.1%	14,742	10.1%
Lung	269	17.0%	17,564	17.9%	70	17.0%	32,425	22.2%
Liver	107	6.8%	7170	7.3%	13	3.2%	8423	5.8%
Prostate	113	7.1%	6105	6.2%	48	11.7%	11,565	7.9%
Cervix, uterus	60	3.8%	2946	3.0%	15	3.6%	5136	3.5%
Ovary	59	3.7%	4514	4.6%	11	2.7%	3623	2.5%
Esophagus	77	4.9%	5505	5.6%	16	3.9%	6693	4.6%
Spleen	85	5.4%	5575	5.7%	14	3.4%	2929	2.0%
Oral	132	8.3%	5430	5.5%	37	9.0%	10,878	7.5%
Thyroid	30	1.9%	3013	3.1%	25	6.1%	6264	4.3%
Lymphoma, leukemia	134	8.5%	8379	8.6%	22	5.3%	8177	5.6%
Bone, soft tissue sarcoma	34	2.1%	1881	1.9%	7	1.7%	2750	1.9%
Brain	55	3.5%	2509	2.6%	5	1.2%	3495	2.4%
Bladder	37	2.3%	2811	2.9%	6	1.5%	3934	2.7%
Testicle	3	0.2%	60	0.1%	3	0.7%	1482	1.0%
Unknown primary	16	1.0%	1211	1.2%	1	0.2%	204	0.1%
Other	142	9.0%	7932	8.1%	36	8.7%	11,986	8.2%
No response	11	0.7%	518	0.5%	20	4.9%	5592	3.8%

^§^Design weight was developed based on the sampling design, with the weight being the inverse of the sampling probability

*SD* standard deviation, *IQR* interquartile range

### Comparison between patients diagnosed with advanced cancer in 2013 and 2016

We compared each item in the newer and earlier diagnosis groups, and the results showed that the two groups had similar trends in most items but differed in some (see details in Table 2). The differences between the two groups were as follows. The mean overall score among patients in the earlier diagnosis group was slightly lower than that in the newer diagnosis group; nonetheless, the difference did not reach the level of statistical significance (Q21, 7.26 vs. 7.47, *P* = 0.19). However, fewer patients in the earlier diagnosis group reported being more satisfied with their treatment selection than did those in the newer diagnosis group (Q13–2, 64.7% vs. 75.8%, *P* < 0.01). The number of respondents who were informed about a second opinion and knew about Cancer Counseling and Support Centers was lower in the earlier diagnosis group than in the newer diagnosis group (Q11, 29.9% vs. 38.4%, *P* = 0.03; Q29, 65.6% vs. 74.3%, *P* = 0.04). In contrast, more respondents in the earlier diagnosis group reported feeling discriminated against because of cancer or experiencing physical distress caused by cancer (Q33–4, 2.4% vs. 5.8%, *P* < 0.01; Q34–2, 58.5% vs. 40.7%, *P* < 0.01).

**Table 2:** Patient experience reports for the newer and earlier diagnosis groups

Questions	Newer diagnosis group (*n* = 1584)	Earlier diagnosis group (*n* = 412)	*P*-value	
Before treatment				
Time from the first consultation to diagnosis was < 1 month (Q8)	70.8%	66.1%	0.23	
Time from diagnosis to the first treatment was < 1 month (Q9)	68.6%	68.9%	0.93	
I was able to talk about cancer or life as a cancer patient with someone after diagnosis (Q10)	79.1%	73.5%	0.11	
My doctor advised me of the possibility of obtaining a second opinion (Q11)	38.4%	29.9%	0.03	*
I received a second opinion (Q12)	22.5%	22.2%	0.95	
I received enough information from medical staff before making treatment decisions (Q13–1)	74.6%	70.9%	0.30	
I am content with my choice of treatment (Q13–2)	75.8%	64.7%	0.00	**
I changed or discontinued treatment owing to financial reasons (Q16)	5.7%	5.3%	0.84	
I altered my financial plans or sought assistance from others to cover my medical expenses (Q17)	36.1%	29.4%	0.05	*
During treatment				
I received enough information about the treatment schedule (Q18–1)	70.6%	72.0%	0.68	
I was able to anticipate the likely side effects of treatment (Q18–2)	61.5%	62.1%	0.88	
I had detailed discussions with medical staff about my treatment (Q18–3)	63.7%	67.3%	0.30	
The medical staff listened and tried to understand my concerns (Q18–4)	70.3%	66.0%	0.25	
My wishes regarding the treatment were respected (Q18–5)	71.6%	69.6%	0.60	
Medical staff responded to my pain or discomfort promptly (Q18–6)	72.8%	75.9%	0.24	
Relevant information was shared among medical staff (Q18–7)	66.3%	68.1%	0.64	
I received treatment from a doctor with expertise (Q18–8)	73.8%	74.0%	0.96	
I felt comfortable talking to the medical staff besides my doctor (Q18–9)	47.9%	48.5%	0.88	
I am satisfied with the treatment I received (Q18–10)	70.6%	72.4%	0.62	
Medical staff offered enough information regarding aspects of daily life while admitted (applicable patients only) (Q18–11)	67.2%	62.4%	0.19	
I visited a referral hospital without any trouble (applicable patients only) (Q18–12)	80.8%	85.7%	0.34	
I was transferred to my preferred hospital (applicable patients only) (Q18–13)	73.5%	79.5%	0.50	
I was asked at every consultation if I had pain during or after treatment (Q19)	78.9%	80.9%	0.58	
I was able to discuss my concerns about the changes in appearance owing to treatment (Q20)	38.2%	40.9%	0.55	
Overall experience from diagnosis to treatment (0–10, average) (Q21)	7.47	7.26	0.19	
I was engaged in paid employment at the time of diagnosis (Q22)	44.9%	56.0%	0.03	*
I told my colleagues about my diagnosis (applicable patients only) (Q23)	81.2%	80.7%	0.92	
My colleagues considered and managed the situation so that I could keep working while receiving treatment (applicable patients only) (Q24)	73.5%	59.2%	0.03	*
I utilized existing resources to balance my treatment and work (applicable patients only) (Q25)	31.8%	39.0%	0.30	
I received some advice from the medical staff about continuing to work (applicable patients only) (Q26)	39.6%	32.9%	0.30	
I resigned or closed business owing to treatment (applicable patients only) (Q27–1–1)	30.0%	37.0%	0.30	
I took a leave of absence but did not resign or close business (applicable patients only) (Q27–1–2)	48.6%	42.4%	0.20	
After treatment				
I feel that cancer treatment for the general public has improved compared to a few years ago (Q28–1)	73.0%	71.4%	0.59	
I feel that there is sufficient support, services, and places for cancer patients and their families to discuss their concerns about cancer (Q28–2)	47.2%	45.6%	0.64	
I am aware of cancer counseling and support centers (Q29)	74.3%	65.6%	0.04	*
I am aware of peer support (Q30)	29.5%	32.8%	0.42	
I know what clinical trials are (Q31)	45.1%	42.1%	0.55	
I am aware of genome-based cancer treatments (Q32)	20.3%	17.7%	0.42	
I am a burden on my family because of my cancer (patients only) (Q33–1)	60.8%	52.2%	0.12	
I am a burden on people outside of my family because of my cancer (patients only) (Q33–2)	32.2%	25.3%	0.06	
I received too much unnecessary attention after my cancer diagnosis (patients only) (Q33–3)	21.1%	15.9%	0.06	
I feel discriminated against by people outside of my family because I have cancer (patients only) (Q33–4)	5.8%	2.4%	0.00	**
I am able to consult with medical staff when feeling pain or discomfort (patients only) (Q33–5)	52.0%	50.4%	0.79	
I am able to consult with medical staff when experiencing mental distress (patients only) (Q33–6)	33.0%	32.6%	0.94	
I am able to go about my daily life now (patients only) (Q33–7)	63.6%	72.4%	0.03	*
I have sufficient support to relieve my physical pain and mental distress (patients only) (Q34–1)	44.2%	44.1%	0.99	
I have no physical distress caused by cancer or cancer treatment (patients only) (Q34–2)	40.7%	58.5%	0.01	**
I have no pain caused by cancer or cancer treatment (patients only) (Q34–3)	59.2%	71.1%	0.03	*
I have no mental distress owing to cancer or cancer treatment (patients only) (Q34–4)	49.6%	60.0%	0.08	
I have no difficulties going about my daily life owing to pain and discomfort from cancer or cancer treatment (patients only) (Q34–5)	55.1%	67.5%	0.05	

Percentages represent the proportion of positive responses for each question. Statistically significant differences are indicated (*P < 0.05; **P < 0.01). Results are unavailable for Q14–15 owing to a small sample size

### Analysis of patient and proxy responses

Stratified analyses according to respondent type are presented in Supplementary Tables 1 and 2. The responses of the patients themselves were not significantly different for most items between the groups. The exceptions were better experiences reported by patients in the earlier diagnosis group than those in the newer diagnosis group, with greater proportions reporting no trouble in referral (Q18-12, 96.9% vs. 86.5%, *P* = 0.02), being transferred to a preferred hospital (Q18-13, 97.5% vs. 84.5%, *P* = 0.01), being able to go about daily life (Q33-7, 72.4% vs. 63.6%, *P* = 0.03), and no physical distress (Q34-2, 58.5% vs. 40.7%, *P* = 0.01) or pain (Q34-3, 71.1% vs. 59.2%, *P* = 0.03). Furthermore, among patients in the earlier diagnosis group, 2.4% reported feeling discriminated against, compared to 5.8% in the newer diagnosis group (*P* < 0.01). However, when we compared the proxy responses, the earlier diagnosis group had significantly less favorable responses in 8 out of 38 items compared with those in the newer diagnosis group.

## Discussion

We assessed cancer patients’ experiences across different diagnosis years using PES data. Our results indicate that survey responses from patients diagnosed 6 years prior were generally less favorable regarding access to treatment-related information, overall care assessment, and post-treatment experience compared with those from more recently diagnosed patients. However, stratified analysis according to respondent type showed that negative feedback was more prevalent in proxy responses than in patient responses, with a higher proportion of proxy responses observed in the earlier diagnosis group. To our knowledge, this is the first nationwide study to investigate differences in responses from patients in Japan 3 and 6 years after diagnosis.

These findings are consistent with those of previous studies. Long-term breast and colorectal cancer patients generally reported good overall QoL scores [4, 5]. Our results showed that more patients diagnosed 6 years prior felt that they were able to go about their daily lives than patients diagnosed 3 years prior. Furthermore, physical and psychological symptoms related to cancer or cancer treatment were relatively few among those who were diagnosed in 2013. These findings are consistent with those of previous studies [6]. Conversely, approximately 30–40% of patients diagnosed in 2013 experienced physical or mental distress. Our results suggest that most long-term survivors cope with their diseases better over time, resulting in more positive experiences compared with those diagnosed later. However, it is important to note that some patients still experience cancer symptoms, treatment-related adverse effects, or social prejudice. The needs of cancer patients change over time, and we must carefully assess their needs and voices at the right time to comprehend their challenges.

Previous studies have indicated that patients with advanced cancer often have complex experiences characterized by a mixture of positive and negative aspects. Our research indicated that most items did not show differences across the years of diagnosis. However, respondents from the earlier diagnosis group provided more favorable responses than those from the newer diagnosis group for a few items. Individuals with advanced-stage cancer strive to alleviate their suffering and seek significance in life by engaging in internal reflection and processing [7]. The results of our study reflect that patients with cancer experience various types of distress, including mental and financial distress, regardless of the duration since their cancer diagnosis. Healthcare personnel should carefully communicate with patients and family members and provide sufficient information to ease anxiety related to cancer or cancer treatment [22].

When surveying patient experiences, it is inevitable to ask proxies for those who cannot answer the questionnaire themselves because of death or disease conditions. Proxy responses are generated to reflect how the patient might think or feel about the situation if they were capable. These responses can serve as substitutes for evaluating the cancer experience, providing a useful measure, particularly when patients are unable to respond owing to cognitive impairment or mortality [23]. In our study, the survey of patients diagnosed in 2013 had a higher proxy response rate than that of patients diagnosed in 2016 (43.2% vs. 38.0%). This may be because more patients died in the earlier diagnosis group than in the newer diagnosis group. Nonetheless, a proxy response may result in a biased representation of the patient’s perspective [23]. However, cancer affects not only the patients themselves but also their families or surroundings. Our study revealed that fewer proxies felt there was sufficient support, services, and places in the earlier diagnosis group compared to the newer diagnosis group (29.6% vs. 46.7%, *P* < 0.01). The overall experience was less favorable in proxy responses from both groups than in responses by the patients themselves; however, responses to most of the items did not differ much between self-responses and proxy responses. Our finding that proxy responses differed only slightly from self-responses is consistent with the findings of previous research [24]. The present study suggests that self-responses and proxy responses may need to be evaluated separately depending on the question types or the proportion of proxy responses.

This study had some limitations that must be considered when interpreting the results. First, the response rate was lower among patients diagnosed 6 years prior than among those diagnosed 3 years prior. Some patients may have been unable to answer questions owing to disease progression or having relocated, resulting in the questionnaire not reaching them. Second, our results may have been influenced by selection bias. Individuals who responded to our survey may have been in better health and/or had stronger opinions about cancer or cancer treatment than those who did not respond. Third, misclassifications in the cancer stage of patients diagnosed in 2016 may have occurred because the cancer stage in this group was based on a self-administered questionnaire. Finally, issues related to multiple testing persisted in our study, potentially elevating the risk of type I errors and false positives. Despite these challenges, our study provided some insights into the mechanisms behind varying results across different survey timings using representative samples from Japan.This study provides insights into the experiences of cancer patients in Japan, emphasizing the importance of survey timing and the role of proxy responses. Cancer poses a substantial burden on patients and their families, especially in cases of advanced-stage cancer and long-term survival. To effectively support cancer patients, capturing patient experiences is crucial for understanding their needs, which, in turn, informs the planning of appropriate approaches in cancer control policies. Differences in patient-reported experiences across diagnosis years were minimal; instead, the variations observed were largely due to the increased reliance on proxy responses in the earlier diagnosis group. These findings underscore the necessity of tailored communication and support for proxies, alongside direct patient care. Policymakers should incorporate these results into cancer control strategies to better address the evolving needs of patients and families managing advanced-stage cancer and long-term survivorship.

## Supplementary Information

Below is the link to the electronic supplementary material.

Supplementary file1 (DOCX 332 KB)

## Data Availability

The datasets generated and/or analyzed during the current study are not made publicly available to protect the confidentiality of participants but are available from the corresponding author on reasonable request.
