# Supplementary material for: Differences in experiences of patients with advanced cancer in Japan from 3 to 6 years after diagnosis
Source: J Cancer Surviv. 2025 Feb 13;20(4):1622–30. doi: 10.1007/s11764-025-01761-0 (PMC13375664; doi:10.1007/s11764-025-01761-0)
Supplement: Supplementary file 1 — Supplementary file1 (DOCX 332 KB) [file 11764_2025_1761_MOESM1_ESM.docx]

Supplementary Information

Journal: *Journal Cancer of Survivorship*

Title: Differences in Experiences of Patients with Advanced Cancer in Japan from 3 to 6 Years after Diagnosis

Yuichi Ichinose^1,2^, Tsutomu Toida^3^, Tomone Watanabe^1,2^, Takafumi Wakita^4^, Takahiro Higashi^1,2^

^1^ Department of Public Health and Health Policy, Graduate School of Medicine, The University of Tokyo, Hongo 7-3-1 Bunkyo-ku, Tokyo 113-0033, Japan

^2^ Division of Health Services Research, Institute for Cancer Control, National Cancer Center, Tsukiji 5-1-1 Chuo-ku, Tokyo 104-0045, Japan

^3^Faculty of Economics, Dokkyo University, Gakuen-cho 1-1 Soka-shi, Saitama 340-0042, Japan

^4^ Faculty of Sociology, Kansai University, Yamate-cho 3-3-35 Suita-shi, Osaka 564-8680, Japan

Corresponding Author: Yuichi Ichinose

Department of Public Health and Health Policy,

Graduate School of Medicine, The University of Tokyo,

Hongo 7-3-1 Bunkyo-ku, Tokyo 113-0033, Japan

E-mail: yichinose@g.ecc.u-tokyo.ac.jp

Tel: 81-3-3812-2111

Contents

# Supplementary Table 1. Patient responses in the newer and earlier diagnosis groups

# Supplementary Table 2. Proxy responses in the newer and earlier diagnosis groups

# Supplementary Fig. 1. Stratified two-stage random sampling of the Patient Experience Survey

Supplementary Table 1. Patient responses in the newer and earlier diagnosis groups

| **Questions** | **Newer diagnosis group**  **(n = 987)** | **Earlier diagnosis group**  **(n = 223)** | ***P*-value** |  |
| --- | --- | --- | --- | --- |
| **Before treatment** |  |  |  |  |
| Time from the first consultation to diagnosis was < 1 month (Q8) | 71.7% | 66.3% | 0.31 |  |
| Time from diagnosis to the first treatment was < 1 month (Q9) | 68.0% | 64.0% | 0.43 |  |
| I was able to talk about cancer or life as a cancer patient with someone after diagnosis (Q10) | 77.8% | 70.6% | 0.11 |  |
| My doctor advised me of the possibility of obtaining a second opinion (Q11) | 41.0% | 31.3% | 0.07 |  |
| I received a second opinion (Q12) | 22.0% | 21.8% | 0.96 |  |
| I received enough information from medical staff before making treatment decisions (Q13–1) | 78.7% | 75.3% | 0.39 |  |
| I am content with my choice of treatment (Q13–2) | 82.7% | 72.9% | 0.03 | * |
| I changed or discontinued treatment owing to financial reasons (Q16) | 3.9% | 6.3% | 0.47 |  |
| I altered my financial plans or sought assistance from others to cover my medical expenses (Q17) | 36.2% | 35.8% | 0.94 |  |
| **During treatment** |  |  |  |  |
| I received enough information about the treatment schedule (Q18–1) | 79.9% | 79.3% | 0.90 |  |
| I was able to anticipate the likely side effects of treatment (Q18–2) | 67.6% | 62.6% | 0.39 |  |
| I had detailed discussions with medical staff about my treatment (Q18–3) | 68.1% | 73.7% | 0.23 |  |
| The medical staff listened and tried to understand my concerns (Q18–4) | 75.5% | 73.2% | 0.61 |  |
| My wishes regarding the treatment were respected (Q18–5) | 77.0% | 72.5% | 0.30 |  |
| Medical staff responded to my pain or discomfort promptly (Q18–6) | 79.4% | 82.8% | 0.27 |  |
| Relevant information was shared among medical staff (Q18–7) | 71.8% | 75.6% | 0.32 |  |
| I received treatment from a doctor with expertise (Q18–8) | 81.8% | 83.2% | 0.69 |  |
| I felt comfortable talking to the medical staff besides my doctor (Q18–9) | 52.7% | 52.5% | 0.97 |  |
| I am satisfied with the treatment I received (Q18–10) | 82.0% | 86.5% | 0.16 |  |
| Medical staff offered enough information regarding aspects of daily life while admitted (applicable patients only) (Q18–11) | 74.8% | 75.6% | 0.87 |  |
| I visited a referral hospital without any trouble (applicable patients only) (Q18–12) | 86.5% | 96.9% | 0.02 | * |
| I was transferred to my preferred hospital (applicable patients only) (Q18–13) | 84.5% | 97.5% | 0.01 | ** |
| I was asked at every consultation if I had pain during or after treatment (Q19) | 75.4% | 75.1% | 0.96 |  |
| I was able to discuss my concerns about the changes in appearance owing to treatment (Q20) | 40.5% | 42.0% | 0.83 |  |
| Overall experience from diagnosis to treatment (0–10, average) (Q21) | 8.07 | 8.12 | 0.69 |  |
| I was engaged in paid employment at the time of diagnosis (Q22) | 51.5% | 59.3% | 0.17 |  |
| I told my colleagues about my diagnosis (applicable patients only) (Q23) | 83.8% | 86.6% | 0.60 |  |
| My colleagues considered and managed the situation so that I could keep working while receiving treatment (applicable patients only) (Q24) | 75.3% | 55.9% | 0.07 |  |
| I utilized existing resources to balance my treatment and work (applicable patients only) (Q25) | 37.4% | 36.3% | 1.00 |  |
| I received some advice from the medical staff about continuing to work (applicable patients only) (Q26) | 40.3% | 41.2% | 0.92 |  |
| I resigned or closed business owing to treatment (applicable patients only) (Q27–1-1) | 27.8% | 32.1% | 0.60 |  |
| I took a leave of absence but did not resign or close business (applicable patients only) (Q27–1-2) | 52.4% | 45.9% | 0.51 |  |
| **After treatment** |  |  |  |  |
| I feel that cancer treatment for the general public has improved compared to a few years ago (Q28–1) | 79.4% | 81.0% | 0.81 |  |
| I feel that there is sufficient support, services, and places for cancer patients and their families to discuss their concerns about cancer (Q28–2) | 47.8% | 64.0% | 0.06 |  |
| I am aware of cancer counseling and support centers (Q29) | 77.5% | 72.2% | 0.34 |  |
| I am aware of peer support (Q30) | 31.7% | 36.1% | 0.30 |  |
| I know what clinical trials are (Q31) | 45.5% | 50.1% | 0.94 |  |
| I am aware of genome-based cancer treatments (Q32) | 19.7% | 26.4% | 0.68 |  |
| I am a burden on my family because of my cancer (patients only) (Q33–1) | 60.8% | 52.2% | 0.12 |  |
| I am a burden on people outside of my family because of my cancer (patients only) (Q33–2) | 32.2% | 25.3% | 0.06 |  |
| I received too much unnecessary attention after my cancer diagnosis (patients only) (Q33–3) | 21.1% | 15.9% | 0.06 |  |
| I feel discriminated against by people outside of my family because I have cancer (patients only) (Q33–4) | 5.8% | 2.4% | 0.00 | ** |
| I am able to consult with medical staff when feeling pain or discomfort (patients only) (Q33–5) | 52.0% | 50.4% | 0.79 |  |
| I am able to consult with medical staff when experiencing mental distress (patients only) (Q33–6) | 33.0% | 32.6% | 0.94 |  |
| I am able to go about my daily life now (patients only) (Q33–7) | 63.6% | 72.4% | 0.03 | * |
| I have sufficient support to relieve my physical pain and mental distress (patients only) (Q34–1) | 44.2% | 44.1% | 0.99 |  |
| I have no physical distress caused by cancer or cancer treatment (patients only) (Q34–2) | 40.7% | 58.5% | 0.01 | ** |
| I have no pain caused by cancer or cancer treatment (patients only) (Q34–3) | 59.2% | 71.1% | 0.03 | * |
| I have no mental distress owing to cancer or cancer treatment (patients only) (Q34–4) | 49.6% | 60.0% | 0.08 |  |
| I have no difficulties going about my daily life owing to pain and discomfort from cancer or cancer treatment (patients only) (Q34–5) | 55.1% | 67.5% | 0.05 |  |

Percentages represent the proportion of positive responses for each question. Statistically significant differences are indicated (*, P<0.05; **, P<0.01). Results are unavailable for Q14–15 owing to a small sample size.

Supplementary Table 2. Proxy responses in the newer and earlier diagnosis groups

| **Questions** | **Newer diagnosis group**  **(n = 590)** | **Earlier diagnosis group**  **(n = 188)** | ***P*-value** |  |
| --- | --- | --- | --- | --- |
| **Before treatment** |  |  |  |  |
| Time from the first consultation to diagnosis was < 1 month (Q8) | 69.0% | 65.8% | 0.47 |  |
| Time from diagnosis to the first treatment was < 1 month (Q9) | 69.8% | 75.5% | 0.27 |  |
| I was able to talk about cancer or life as a cancer patient with someone after diagnosis (Q10) | 81.7% | 77.6% | 0.43 |  |
| My doctor advised me of the possibility of obtaining a second opinion (Q11) | 33.7% | 27.8% | 0.25 |  |
| I received a second opinion (Q12) | 23.6% | 22.7% | 0.89 |  |
| I received enough information from medical staff before making treatment decisions (Q13–1) | 67.4% | 64.9% | 0.66 |  |
| I am content with my choice of treatment (Q13–2) | 63.6% | 53.9% | 0.10 |  |
| I changed or discontinued treatment owing to financial reasons (Q16) | 8.3% | 4.0% | 0.07 |  |
| I altered my financial plans or sought assistance from others to cover my medical expenses (Q17) | 36.1% | 20.8% | 0.00 | ** |
| **During treatment** |  |  |  |  |
| I received enough information about the treatment schedule (Q18–1) | 54.7% | 61.6% | 0.24 |  |
| I was able to anticipate the likely side effects of treatment (Q18–2) | 51.3% | 61.2% | 0.03 | * |
| I had detailed discussions with medical staff about my treatment (Q18–3) | 56.1% | 58.8% | 0.56 |  |
| The medical staff listened and tried to understand my concerns (Q18–4) | 61.5% | 55.8% | 0.46 |  |
| My wishes regarding the treatment were respected (Q18–5) | 62.6% | 65.4% | 0.71 |  |
| Medical staff responded to my pain or discomfort promptly (Q18–6) | 61.7% | 66.5% | 0.32 |  |
| Relevant information was shared among medical staff (Q18–7) | 57.2% | 57.4% | 0.97 |  |
| I received treatment from a doctor with expertise (Q18–8) | 60.0% | 61.3% | 0.85 |  |
| I felt comfortable talking to the medical staff besides my doctor (Q18–9) | 40.1% | 43.1% | 0.61 |  |
| I am satisfied with the treatment I received (Q18–10) | 51.3% | 51.9% | 0.92 |  |
| Medical staff offered enough information regarding aspects of daily life while admitted (applicable patients only) (Q18–11) | 55.7% | 46.4% | 0.13 |  |
| I visited a referral hospital without any trouble (applicable patients only) (Q18–12) | 78.2% | 76.4% | 0.82 |  |
| I was transferred to my preferred hospital (applicable patients only) (Q18–13) | 68.1% | 66.0% | 0.87 |  |
| I was asked on every consultation if I had pain during or after treatment (Q19) | 86.5% | 90.9% | 0.20 |  |
| I was able to discuss my concerns about the changes in appearance owing to treatment (Q20) | 34.7% | 39.6% | 0.32 |  |
| Overall experience from diagnosis to treatment (0–10, average) (Q21) | 6.5 | 6.1 | 0.28 |  |
| I was engaged in paid employment at the time of diagnosis (Q22) | 34.0% | 51.7% | 0.00 | ** |
| I told my colleagues about my diagnosis (applicable patients only) (Q23) | 74.7% | 70.9% | 0.71 |  |
| My colleagues considered and managed the situation so that I could keep working while receiving treatment (applicable patients only) (Q24) | 68.6% | 66.0% | 0.75 |  |
| I utilized existing resources to balance my treatment and work (applicable patients only) (Q25) | 17.2% | 41.7% | 0.01 | ** |
| I received some advice from the medical staff about continuing to work (applicable patients only) (Q26) | 37.3% | 17.0% | 0.01 | * |
| I resigned or closed business owing to treatment (applicable patients only) (Q27–1-1) | 36.1% | 43.3% | 0.41 |  |
| I took a leave of absence but did not resign or close business (applicable patients only) (Q27–1-2) | 38.4% | 35.1% | 0.74 |  |
| **After treatment** |  |  |  |  |
| I feel that cancer treatment for the general public has improved compared to a few years ago (Q28–1) | 62.4% | 56.2% | 0.52 |  |
| I feel that there is sufficient support, services, and places for cancer patients and their families to discuss their concerns about cancer (Q28–2) | 46.7% | 29.6% | 0.00 | ** |
| I am aware of cancer counseling and support centers (Q29) | 69.4% | 61.9% | 0.05 | * |
| I am aware of peer support (Q30) | 26.1% | 27.6% | 1.00 |  |
| I know what clinical trials are (Q31) | 44.3% | 33.4% | 0.24 |  |
| I am aware of genome-based cancer treatments (Q32) | 21.3% | 12.6% | 0.02 | * |

Percentages represent the proportion of positive responses for each question. Statistically significant differences are indicated (*, P<0.05; **, P<0.01). Results are unavailable for Q14–15 owing to a small sample size. Q33–34 were exclusively directed at the patients themselves.

Fig. 1.
